# A retrospective analysis of ultrasound neuromodulation therapy using transcranial pulse stimulation in 58 dementia patients

**DOI:** 10.1017/S0033291725000406

**Published:** 2025-03-04

**Authors:** Sonja Radjenovic, Lena Bender, Martin Gaal, Daria Grigoryeva, Michael Mitterwallner, Sarah Osou, Anna Zettl, Nina Plischek, Patrick Lachmair, Katrin Herzhauser, Eva Matt, Roland Beisteiner

**Affiliations:** 1 Department of Neurology, Medical University of Vienna, Vienna, Austria; 2 Department of Pediatrics and Adolescent Medicine, Medical University of Vienna, Vienna, Austria

**Keywords:** Alzheimer’s disease, brain stimulation, dementia, neuromodulation, transcranial pulse stimulation, ultrasound

## Abstract

**Background:**

Novel ultrasound neuromodulation techniques allow therapeutic brain stimulation with unmet precision and non-invasive targeting of deep brain areas. Transcranial pulse stimulation (TPS), a multifrequency sonication technique, is approved for the clinical treatment of Alzheimer’s disease (AD). Here, we present the largest real-world retrospective analysis of ultrasound neuromodulation therapy in dementia (AD, vascular, mixed) and mild cognitive impairment (MCI).

**Methods:**

The consecutive sample involved 58 patients already receiving state-of-the-art treatment in an open-label, uncontrolled, retrospective study. TPS therapy typically comprises 10 sessions (range 8–12) with individualized MRI-based target areas defined according to brain pathology and individual pathophysiology. We compared the CERAD-Plus neuropsychological test battery results before and after treatment, with the CERAD Corrected Total Score ( CTS) as the primary outcome. Furthermore, we analyzed side effects reported by patients during the treatment period.

**Results:**

CERAD Corrected Total Score (CTS) significantly improved (*p* = .017, *d* = .32) after treatment (Baseline: *M* = 56.56, *SD* = 18.56; Post-treatment: *M* = 58.65, *SD* = 19.44). The group of top-responders (top quartile) improved even by 9.8 points. Fewer than one-third of all patients reported any sensation during treatment. Fatigue and transient headaches were the most common, with no severe adverse events.

**Conclusions:**

The findings implicate TPS as a novel and safe add-on therapy for patients with dementia or MCI with the potential to further improve current state-of-the-art treatment results. Despite the individual benefits, further randomized, sham-controlled, longitudinal clinical trials are needed to differentiate the effects of verum and placebo.

## Background

Transcranial ultrasound neuromodulation (TUS) is a rapidly developing novel brain stimulation technology that has increased interest in clinical research and therapy. TUS allows therapeutic brain stimulation that can directly influence neuronal membrane potentials, potentially opening ion channels and modulating neuronal excitability, or inducing microstreaming and cavitation effects, where oscillating bubbles in the tissue generate shear forces that alter cell behavior and synaptic activity, influencing neurotransmitter release and neuronal network dynamics (Blackmore, Razansky & Götz, 2023; Darmani et al., 2022; Wang et al., 2022). Thus, TUS offers unmet precision and non-invasive targeting of deep brain areas, which is currently not possible with other non-invasive technologies. Moreover, clinical data for several different ultrasound neuromodulation systems have been published (for review see Matt et al., 2024). Available techniques include focused/unfocused and mono−/multifrequency sonication with pulse lengths ranging from the micro- to the millisecond scale (Beisteiner, Lozano, Di Lazzaro, George, & Hallett, 2024). The first patient study applying focused navigated ultrasound was conducted in dementia patients (Beisteiner et al., 2019), yielding significant improvements in CERAD CTS scores following treatment, which persisted for up to 3 months. Moreover, the clinical safety and therapeutic effects for many different diseases have been described, such as chronic pain conditions, psychiatric disorders, disorders of conscientiousness, or movement disorders (for a review, see Beisteiner, Hallett, & Lozano, 2023; Beisteiner et al., 2024; Lee, Weisholtz, Strangman, & Yoo, 2021; Matt et al., 2024; Pellow, Pichardo, & Pike, 2024; Sarica et al., 2022). In addition to prospective clinical studies, analysis of real-world clinical applications represent an important source for determining the clinical utility and tolerability of novel therapies. Owing to variabilities in patient characteristics and patient evaluations via clinical scales, studies including large numbers of patients are warranted.

Here, we present the largest retrospective analysis of real-world ultrasound neuromodulation therapy with a novel multifrequency sonication technique—transcranial pulse stimulation (TPS). TPS is currently the most widely applied ultrasound neuromodulation therapy and is approved for the clinical treatment of Alzheimer’s disease (AD, CE certification [EU] and FDA Investigational Device Exemption [USA]). Data analysis follows our pioneering work (Beisteiner et al., 2019), utilizing a similar sample (dementia patients) and expecting similar results regarding the improvement in the CERAD Corrected Total Score (Ehrensperger, Berres, Taylor, & Monsch, 2010). However, the current retrospective analysis involves a larger sample of dementia and MCI patients, assessing the utility of the same therapeutic method in clinical practice and as an add-on therapy, alongside patients’ running state-of-the-art treatment.

## Materials and methods

### Patients

The inclusion criterion for retrospective analysis was a patient’s request for an add-on treatment with the novel, well-tolerated ultrasound treatment. Data were recorded at the TPS Therapy and Development Centre in Vienna (Austria) after written informed consent was obtained. In this study, we included 58 first-time treated patients with completed CERAD CTS pre/post data (male: 32; female: 26; age range: 52–82; *M* = 71.72, *SD* = 8.19). Most patients presented with a diagnosis of AD (*N* = 41), some with mild cognitive impairment (MCI) (*N* = 5), vascular dementia (*N* = 2), or mixed dementia (*N* = 10). All patients received their diagnoses from individual neurologists as a part of normal clinical care. Patients were included based on the written diagnosis from the referring physician. Procedural data about the establishment of the diagnoses by the individual neurologists was not available. This procedure corresponds to the goal of investigating possible therapeutic add-on effects in a real-life clinical care cohort as opposed to a highly selected study cohort. The amount of sessions was mostly 10, with one patient having eight, and one twelve sessions. For more detailed patient characteristics (such as individual medication, comorbidities, stimulated brain regions, or individual values in the primary outcome) (see Supplemental file).

### Study design

This was an open-label, uncontrolled, retrospective study on the clinical add-on effects of therapeutic transcranial ultrasound performed with TPS (NEUROLITH TPS system, Storz Medical AG, Tägerwilen, Switzerland) (Beisteiner et al., 2019; Radjenovic, Dörl, Gaal, & Beisteiner, 2022). Before treatment, previous and current clinical diagnoses, medications, and possible contraindications were assessed according to the guidelines of the manufacturer (intracerebral bleeding, thrombosis, pregnancy, tumor in the treatment area, cortisone treatment up to 6 weeks before the start of the treatment, metal objects in the head, and non-approved pacemakers). Patients were instructed not to change their usual medication and treatment settings during the time of TPS therapy. Before beginning treatment, high-resolution magnetic resonance imaging (MRI) was conducted to assist with individualized treatment planning, target specific brain regions, and rule out contraindications with respect to brain morphology and pathology. Patients then underwent navigated individualized TPS therapy, which is essential in the setting of patient-specific clinical neuromodulation (Beisteiner et al., 2024). One ultrasound neuromodulation session lasted 30 to 45 minutes. Highly individualised treatments were performed according to clinical state, brain pathology, and individual pathophysiology (for details, see Supplemental file) (Beisteiner et al., 2024). The target areas were selected based on Beisteiner et al. (2019), including the bilateral parietal and frontal regions, and occipital/temporal regions, but individually adapted and extended to deep brain areas (anterior cingulate cortex, hippocampus, and precuneus) (Supplemental file). The typical treatment course included 2000–4000 ultrashort (~3 μs) ultrasound pulses per session (energy flux density = 0.15–0.25 mJ/mm^2^ and pulse repetition rate = 4 Hz), with precise targeting based on individual patients’ MR scans. Cognitive assessment was routinely conducted a few days before and after treatment by clinical neurologists or clinical psychologists selected by the patients and their caregivers. We investigated the following research questions: (1) Are there indications of therapeutic add-on effects based on the primary outcome, the CERAD Corrected Total Score?; and (2) Is the therapy safe and feasible in dementia patients in a clinical setting?

### Assessments

#### Primary outcome

*Consortium to Establish a Registry for Alzheimer’s Disease (CERAD-Plus).* The CERAD test battery (developed by the National Institute on Aging in 1986) is a pen-and-paper cognitive assessment including nine different tasks on memory, language, praxis, and orientation (see Table 2 for specific tasks). The CERAD-Plus includes three additional tasks for better assessment of subcortical disorders: TMT-A (psychomotor speed), TMT-B (executive functions), and Word Fluency S-words (frontal executive functions) (Schmid, Ehrensperger, Berres, Beck, & Monsch, 2014) For the purpose of this study, we used the CERAD CTS, which is an age-, sex-, and education level-corrected score that provides an overall measure of cognitive function.

#### Side effects and adverse events (AE)

*Patient safety evaluation, side effects, and adverse events.* Self-developed scales were used to quantify pain and pressure sensations during therapy. The subjective patients’ ratings ranged from 0 = *no pain/pressure* to 10 = *maximal pain/pressure.* These were assessed for each therapy session individually. In addition, in an open-format protocol, patients indicated whether they had experienced any other symptoms over the treatment period. These were all considered side effects (i.e., symptoms known to be directly linked to the therapy, as described by Radjenovic et al., 2022). The severity of these side effects was graded using the Common Terminology Criteria for Adverse Events (CTCAE, v. 5.0, 2017) on a scale from 1 (mild) to 3 (severe).

#### Data analytical strategy

We aimed to compare the pre- and posttreatment results of the cognitive assessments and evaluate patient safety by quantifying pain and pressure sensations during treatment as well as side effects during the period of treatment.

We conducted descriptive analyses (minimum, maximum, mean, standard deviation) and two-sided paired *t*-tests (with a significant *p*-value of < .05) for the primary outcome CERAD CTS. In addition, a principal component analysis (PCA) was applied to extract the most important factors of the CERAD-Plus. For further analysis regarding the logistic regression score (LR, [Ehrensperger et al., 2010] *N* = 48) and the PCA score (*N* = 46), data corrected for age, gender, and formal education were generated through z-transformation (as performed by the CERAD Online analysis; norm population CERAD: *N* = 1100, phonemic word fluency: *N* = 604). The LR score weights those CERAD subtests that are particularly indicative of AD-type dementia (Bessi et al., 2018).

With respect to the exploratory PCA, we followed our previous publication (Beisteiner et al., 2019), which also included the CERAD-Plus test battery for its analysis, where we identified relevant CERAD subfactors to determine the factor structure. The Kaiser–Meyer–Olkin measure was used to evaluate the adequacy of the sample for factor analysis (Kaiser, 1970), and Bartlett’s (1950) test of sphericity was employed to check for sufficient significant correlation in the data for factor analysis. Factors with eigenvalues exceeding 1 were identified (Guttman, 1954). A varimax-rotated two-factor solution has been applied to the PCA results, similar to the approach of Ehrensperger et al. (2010) including the phonemic word fluency test. Factors were named based on the tasks with the highest loadings, such as memory, figural, and verbal. PCA, rather than the theory-based approach was used, as it allows the factors to emerge directly from the data and is particularly useful when having patients with complex diseases such as Alzheimer’s, where symptoms can be diverse and multifaceted. Finally, two-sided paired *t*-tests were conducted to assess changes in the identified factors after treatment.

For pain and pressure sensations, means were calculated for each patient over the course of the treatment, and other side effect were noted qualitatively. Statistical analyses were conducted in SPSS v. 29 and R version 4.2.3 (IBM Corp, 2022; R Core Team, 2023).

## Results

### Primary outcome

**Figure 1.**
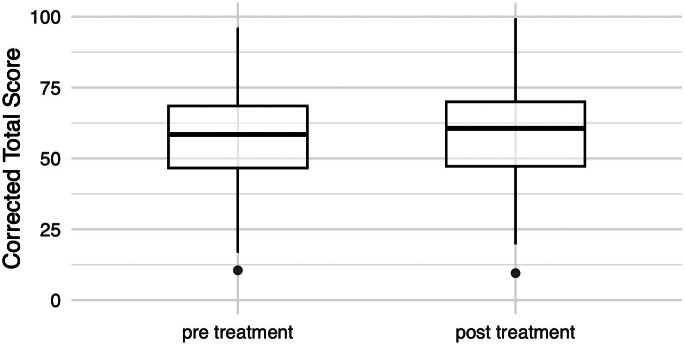
Comparison of the CERAD-Plus CTS pre- and post-TPS treatment *Note.* CTS improved significantly after treatment (two-sided paired t-test, *p* = .017). The bold horizontal line in the middle of the boxplot represents the median, the other two above and underneath it the 25th and the 75th percentiles, whereas the vertical line represents the range of values.

The CERAD Corrected Total Score (CTS), the primary outcome measure, showed a significant improvement following treatment (*p* = .017), with an average increase of 2.10 points, and a small effect size (*d* = .32). For more descriptive analyses, see Table 1 and Figure 1.

**Table 1.** Summary of the results

Descriptive statistics							
	*N*	Min.	Max.		*M*	*SD*	
CTS pre	58	10.52	96.09		56.56	18.56	
CTS post	58	9.52	100.58		58.65	19.44	

*Paired t-tests*							
	*N*	*M*	*SD*	*T*	95% CI (LL; UL)	*p*	*d*
CTS pre-/post-difference	58	2.10	6.47	2.47	0.397; 3.380	.017	.32
LR CTS pre/post difference	48	0.69	2.77	1.73	−0.113; 1.494	.090	.25

*Note: N* varied depending on the type of test, as not all patients provided all the data. LR = logistic regression. CI = confidence interval. Two-sided *p* < .05 significance level.

### Secondary analyses

The exploratory CERAD principal component analysis (PCA) indicated a Kaiser–Meyer–Olkin (1970) measure of .671, representing mediocre factor analysis. The significant Bartlett’s (1951) test of sphericity (*p* < .001) suggested that there was sufficient significant correlation in the data for factor analysis. After the aforementioned initial tests, three factors with eigenvalues exceeding 1 (Guttman, 1954) which accounted for 67.53 of the total variance, were justified empirically and logically. Most tasks showed high loadings on just one of the three factors after the varimax-rotated two-factor solution (a PCA approach similar to that of Ehrensperger et al. (2010) including the phonemic word fluency test). Factor 1 (eigenvalue = 4.53, explained variance = 41.15%) was named VERBAL, as its highest loadings were found for the verbal fluency tasks and the naming task. Factor 2 (eigenvalue = 1.69, explained variance = 15.39%) was named FIGURAL, as it yielded the highest loadings on the figural tasks. Factor 3 (eigenvalue = 1.21, explained variance = 10.99%) showed the highest loadings on the word list intrusions, delayed recall, and recognition and was thus named MEMORY (see Table 2). However, the *t*-test comparisons revealed no significant changes in any of the factors after treatment (see Table 3). The CERAD logistic regression score (LR), which focuses on tests important for dementia diagnosis, did not improve significantly after treatment according to these data (*N* = 48). For more detailed results, see Table 1.

**Table 2.** The Principal Component Analysis of the CERAD-Plus

Task	PC1	PC2	PC3
Verbal Fluency Animals	**0.907**	0.095	0.060
Boston Naming Test	**0.732**	0.303	−0.176
Word List Memory Total	**0.649**	0.206	0.519
Word List Recall	0.445	0.524	**0.578**
Word List Intrusions	−0.190	−0.037	**0.793**
Word List Savings (%)	−0.075	**0.597**	0.247
Word List Recognition Discriminability (%)	0.285	0.398	**0.532**
Constructional Praxis	0.339	**0.502**	−0.016
Recall of Constructional Praxis	0.215	**0.901**	0.076
Constructional Praxis Savings (%)	0.151	**0.867**	0.038
Phonemic Fluency S-words	**0.876**	0.061	0.066

*Note.* Extraction method: Principal component analysis; rotation method: varimax with Kaiser normalization. The highest loadings are in bold.

**Table 3.** The results of the paired t-tests concerning the principal component analysis (PCA) of the CERAD-Plus

Factor	*N*	*M*	*SD*	*T*	95% CI (LL; UL)	*p*	*d*
VERBAL	46	0.06	0.56	0.71	−0.108; 0.225	.483	.10
FIGURAL	45	0.14	0.77	1.23	−0.090; 0.372	.225	.18
MEMORY	46	0.11	0.72	1.09	−0.098; 0.327	.283	.16

*Note. N* varied, as not all patients provided all the data. CI = confidence interval. Two-sided *p* < .05 significance level.

We furthermore analyzed the characteristics of the top responders to treatment. As no standard for the Minimal Clinically Important Difference (MCID) or top responders in CERAD Plus exists (e.g., Falkenreck et al., 2023), we adopted a data-driven approach using percentile distribution. Specifically, we defined top responders as patients above the third quartile (Q3) of improvement scores, corresponding to the top 25% (*N* = 15). The findings revealed that most of these patients were men (66.67%), and had AD diagnosis (46.67%; of which mostly incipient form of AD), or MCI (33.33%). The CERAD-corrected total score (CTS), the primary outcome measure, showed a significant improvement following treatment (*p* < .001), with an average increase of 9.80 points, and a large effect size (*d* = 4.97). For the pre- and post-CTS , as well as other characteristics, including age and education – which showed no distinct shared specifics and are similar to the original sample findings – see Table 4.

**Table 4.** The characteristics of the best treatment responders

	Min.	Max.	*M*	*SD*
Age	54	82	72.80	8.63
Education	8	20	11.73	2.96
CTS (pre)	35.84	92.58	60.97	15.67
CTS (post)	43.84	100.58	70.77	16.32

*Paired t-test*						
	*M*	*SD*	*T*	95% CI (LL; UL)	*p*	*d*
CTS pre/post difference	9.80	1.97	19.26	8.708; 10.891	<.001	4.97

*Note. N* = 15 (top 25% of treatment responders).

### Side effects

Among the 58 patients, 22.4% (*M* = 2.5 [scale of 1–10]) reported pressure sensations, and 25.9% (*M* = 2.2 [scale of 1–10]) reported pain sensations during the 10 therapy sessions. Following the temporal trend analysis, we observed a decrease in both pain and pressure scores over the treatment period, however, these changes were not statistically significant. This lack of significance may be attributed to the generally low baseline scores on the pain and pressure scales. Furthermore, across a total of 570 treatment sessions (involving 57 patients, with one patient missing data), no sensations, or side effects were reported in 81.40% of the sessions. The most common were fatigue (6.84% of total therapy sessions), transient pain (mostly headaches) (4.21% of total therapy sessions), and pressure sensations (2.98% of total therapy sessions). Other, such as dizziness, nausea, confusion, and gait disturbance were reported to a lesser extent (< 3%). Side effects were transient and infrequent with regard to the number of sessions per patient (Supplemental file). There was not sufficient data on the intensity available (due to an open-form format protocol) yielding unquantifiable data. The severity of all reported side effects was defined as 1 (mild), as no medical interventions were needed and no new or additional limitations in daily activities were reported by patients or caregivers.

## Discussion

This is currently the largest retrospective analysis of real-world ultrasound neuromodulation therapy focused on clinical add-on effects in patients already on state-of-the-art treatment. Fifty-eight patients suffering from dementia or MCI were analyzed for cognitive improvements and possible side effects or adverse events following TPS treatment. The results revealed significant cognitive improvement as a therapeutic add-on effect of TPS ultrasound neuromodulation (based on the CERAD-Plus CTS).

In previous studies, ultrasound neuromodulation demonstrated an excellent safety profile for humans (Lee et al., 2021; Radjenovic et al., 2022; Legon et al., 2020; Pasquinelli, Hanson, Siebner, Lee, & Thielscher, 2019). but requires specific clinical neuroscientific expertise for therapeutic application in brain diseases (Pellow et al., 2024, Beisteiner et al. 2024). With adequate application, various ultrasound neuromodulation techniques are well tolerated, with no serious or lasting side effects, adverse events, or morphological brain changes reported. As expected, no moderate or severe side effects or adverse events were found in this study, with a few patients experiencing low-intensity pain and pressure sensations during treatment. Across the treatment period, the most common were fatigue, transient pain (mainly headaches), and pressure sensations. Overall, in this real-world ultrasound neuromodulation cohort, the TPS appears safe and feasible for dementia or MCI patients.

In one of our prior studies (Beisteiner et al., 2019) – using a similar yet distinct sample from the current study – both the CERAD-Plus CTS and logistic regression score (LR) significantly improved after treatment and were maintained for 3 months. In contrast, our current study, which lacked follow-up data, revealed significant but less pronounced improvements in the CTS and a lack of significantly improved LR values. These differences are most likely due to considerably greater interpatient variability, with more advanced cases (i.e., patients in later stages of the disease or patients with comorbidities) requesting a therapeutic TPS trial. We hypothesize that a better biological brain state (i.e., patients in earlier stages of the disease or patients with fewer comorbidities) is compatible with better neuroplastic reorganization capabilities, resulting in a greater probability of treatment response (Matt et al., in press). Previously published ultrasound neuromodulation studies with considerably fewer patient samples suffering from dementia/MCI reported variable effect sizes. Cont et al. (2022) and Fong et al. (2023) reported medium to large effects, whereas Shimokawa et al. (2022) reported no significant improvements in any of the cognitive tests after treatment.

Considerable variability is also inherent in electromagnetic brain stimulation (NIBS) techniques such as transcranial magnetic stimulation (TMS) and transcranial direct current stimulation (tDCS). Meta-analyses of patients with various clinical diagnoses (dementia, among others) revealed small effects on working memory and attention/vigilance (TMS, tDCS; Begemann, Brand, Ćurčić-Blake, Aleman, & Sommer, 2020) substantial effects on global cognition in AD and MCI patients with high-frequency rTMS (Teselink et al., 2021) and immediate moderate effects on memory function not persistent at the 1-month follow-up (rTMS) (Chu et al., 2021). The latter meta-analysis additionally revealed significant impairment in memory function at the 1-month follow-up after atDCS, with a large effect size reported. Regarding the clinical variability of non-invasive electromagnetic technologies, it is important to note that electromagnetic brain stimulation provides limited targeting options within pathological brains (due to field distortions) and that there is no possibility for deep brain stimulation (Mantell et al., 2021). Althogether, available data argue for more studies with larger patient samples within the field of neuromodulation therapy.

Regarding already established dementia therapies, medical treatment showed a small to medium treatment effect on cognition and global, functional, and behavioral scales in Alzheimer’s disease patients (e.g., Smith, Wells & Borrie, 2006). However, medical treatment can often be accompanied by adverse events, for example, a two- to fivefold higher risk of gastrointestinal, neurological, and cardiovascular complications, with the most severe including weight loss, debility, and syncope (Buckley & Salpeter, 2015). Cognitive training moderately enhanced global cognition, memory, and attention in mild cognitive impairment, however, the amount of benefit for dementia is under debate (Hill et al., 2017). Larger studies are needed to better understand the differential treatment effects of the various dementia therapies, and possible placebo effects need to be considered (Osou et al., 2024).

Importantly, our sample represents patients requesting ultrasound neuromodulation as an add-on therapy in a private medical institution. In such real-world data, large inter-patient variability is inevitable due to the diverse patient population, including comorbidities. While such cohorts offer valuable insights into the effects of ultrasound neuromodulation in real-world settings, they also present a challenge: the large inter-patient variability makes it difficult to determine whether the treatment can consistently induce positive changes across a broader, heterogeneous group. Please note, that patients were requested to keep all running therapies stable during the TPS add-on intervention, thus co-therapeutic inferences were minimized.

## Conclusions

We conclude that findings from the currently largest retrospective analysis of real-world ultrasound neuromodulation therapy implicate TPS as a novel and safe add-on therapy. These findings are particularly important given the rapidly increasing number of individuals suffering from memory impairment. TPS has the potential to further improve current state-of-the-art treatment results. Despite the individual benefits, further randomized, sham-controlled, longitudinal clinical trials are needed for the differentiation of the effects of verum and placebo.

## Supporting information

Radjenovic et al. supplementary material Radjenovic et al. supplementary material

## Supplementary Material

The supplementary material for this article can be found at http://doi.org/10.1017/S0033291725000406.
