# Supplementary material for: A retrospective analysis of ultrasound neuromodulation therapy using transcranial pulse stimulation in 58 dementia patients
Source: Psychol Med. 2025 Mar 4;55:e70. doi: 10.1017/S0033291725000406 (PMC12080636; doi:10.1017/S0033291725000406)
Supplement: Radjenovic et al. supplementary material [file S0033291725000406sup001.docx]

| Supporting Information Table S1  *Patients’ characteristics, therapy information, individual CTS-Scores, and side effects* | | | | | | | | | | | | | |
| --- | --- | --- | --- | --- | --- | --- | --- | --- | --- | --- | --- | --- | --- |
| **Pat.** | **Gender** | **Age** | **Treatment diagnosis** | **Other Diagnosis** | **Medication** | **Therapy period**  **(in days)** | **Num. of sessions** | **Stimulated Brain Regions** | **Days between CERAD-Plus (pre) and first TPS session** | **Days between last TPS session and CERAD-Plus (post)** | **CTS-Score (pre)** | **CTS-Score (post)** | **Adverse Events (Frequency)** |
| P01 | f | 69 | AD | arterial hypertension, condition after neuroborreliosis, hypercholesterolemi, hysterectomy + anterior and posterior colporrhagia, lumbago, struma nodosa, valgus gonaarthrosis | Rivastigmine 13.3mg/24h, Memantine 20mg | 11 | 10 | parietal bilateral, frontal bilateral, precuneus, anterior cingulum | 13 | 6 | 30.49 | 36.49 | Fatigue  (1/10 sessions) |
| P02 | m | 77 | MCI | condition after fract. ossis metros dext., condition after traumatic brain injury | Cinnarizine 80mg | 11 | 10 | parietal bilateral, frontal bilateral, precuneus, anterior cingulum | 0 | 12 | 79.94 | 90.94 | pain (1/10 sessions) |
| P03 | 1 | 78 | AD | condition after femoral neck fracture, osteoporosis, tonsillectomy, urothelial carcinoma | Rivastigmine 13.3mg/24h, Memantine 20mg | 11 | 10 | parietal bilateral, frontal bilateral, precuneus, anterior cingulum | 18 | 4 | 76.68 | 78.68 | none |
| P04 | m | 77 | Senile dementia of AD type | arterial hypertension, nodus prostatae | Donepezil 10mg, Trospium chloride 15mg | 11 | 12 | parietal ext bilateral, frontal bilateral, precuneus, anterior cingulum | 12 | 5 | 50.04 | 56.04 | confusion (2/10 sessions), fatigue (1/10 sessions) |
| P05 | f | 61 | early-onset AD | none | Rivastigmine 13.3mg/24h | 11 | 10 | parietal bilateral, frontal bilateral, precuneus, occ/temp bilateral | 0 | 0 | 65.00 | 61.00 | none |
| P06 | m | 68 | AD | angiomylipoma, cataract, commotio cerebri, epidurale L4-L5, HLA-B27 positive, osteoartritis | Atorvasstatine 10 mg, Memantine 20mg, Rivastigmine 13.3 mg/24h, Trazodone 100mg | 11 | 10 | parietal bilateral, frontal bilateral, precuneus, occ/temp bilateral | 3 | 0 | 61.23 | 73.23 | fatigue (4/10 sessions), nausea (2/10 sessions) |
| P07 | f | 75 | AD | essential hypertension | Candesartan 8mg, Nimodipine 6mg, Vitamin D3, Acetylsalicylic acid 100mg | 11 | 10 | parietal bilateral, frontal bilateral, precuneus, anterior cingulum | 6 | 6 | 96.09 | 95.09 | NA |
| P08 | f | 68 | incipient AD | Acute posterior hyaloid detachment, Chronic immune thyroiditis, Unconfirmed referral diagnosis Epilepsy, Superficial - multicentric basal cell carcinoma (resection) | Estradiol, Levothyroxine 100mg | 11 | 10 | parietal bilateral, postfrontal bilateral, precuneus, occ/temp bilateral | 10 | 4 | 48.78 | 49.78 | dizziness (1/10 sessions), fatigue (1/10 sessions), nausea (1/sessions), pain (2/10 sessions) |
| P09 | m | 65 | early-onset AD | condition after Morbus Reiter | Rivastigmine 4.6mg | 11 | 10 | parietal bilateral, frontal bilateral, precuneus, anterior cingulum, occ/temp bilateral | 11 | 0 | 38.77 | 35.77 | none |
| P10 | f | 61 | early-onset AD | none | Donepezil 10mg, Escitalopram 20mg, Gingko biloba 240mg, Memantine 20mg | 11 | 10 | parietal bilateral, frontal bilateral, precuneus, anterior cingulum, occ/temp bilateral | 0 | 0 | 10.52 | 9.52 | none |
| P11 | f | 77 | AD | condition after carotid stenosis with surgical repair, condition after cataract surgery, Diabetes mellitus type 2, Hepatopathy of undefined origin, Hyperlipidemia, Osteoporosis, Macular degeneration | Calcium and Vitamin D3 5 00mg/8 00mg, Cinnarizine 80mg, Escitalopram 15mg, Rivastigmine 13.3mg/24h, Trospium chloride 30mg, Lamotrigine 50mg, Metformin 1000mg, Acetylsalicylic acid 100mg | 11 | 10 | parietal bilateral, frontal bilateral, precuneus, cing cort, left occ/tempt, cereb occ | 69 | 52 | 16.77 | 21.77 | none |
| P12 | f | 65 | atypical or mixed dementia | Diabetes mellitus with polyneuropathy, hypertension | Candesartan & Hydrochlorothiazide 8/12.5 (not constantly), Bisoprolol 5mg, Memantine 20mg, Metformin 1000mg, Pantoprazole 20mg, Acetylsalicylic acid 100mg, Olanzapine 2.5-5mg | 11 | 10 | parietal bilateral, frontal bilateral, precuneus, anterior cingulum | 26 | 5 | 72.19 | 70.19 | dizziness (1/10 sessions) |
| P13 | m | 76 | mixed dementia | aetiologically unclear axonal polyneuropathy, gait disturbance | Donepezil 5-10mg | 11 | 10 | parietal + motorcort bilateral, frontal bilateral, precuneus, anterior cingulum | 7 | 3 | 85.41 | 99.41 | dizziness (1/10 sessions), fatigue (3/10 sessions) |
| P14 | m | 82 | MCI with underlying Senile dementia of AD type | benign prostatic hyperplasia, psoriasis vulgaris | Donepezil 10mg, Piracetam, Cinnarizine, Vitamin B | 11 | 10 | parietal ext bilateral, frontal bilateral, precuneus, anterior cingulum | 0 | 0 | 58.49 | 69.49 | none |
| P15 | m | 74 | AD | pain and swelling in the right shoulder | Memantine 20mg, Paracetamol 500mg (long-term medication) | 11 | 10 | parietal bilateral, frontal bilateral, precuneus, anterior cingulum, occ/temp bilateral | 7 | 3 | 49.86 | 60.86 | none |
| P16 | m | 72 | AD | none | Galantamine 16mg | 11 | 10 | parietal biltateral, frontal bilateral, precuneus, anterior cingulum | 0 | 0 | 83.25 | 85.25 | none |
| P17 | m | 67 | AD | acute anterior wall infarction some years ago, aortic ectasia 40mm, coronary heart disease, hypercholesterolemia, prostate hypertrophy | Bisoprolol 5mg, Alirocumab 2x/Monat, Rivastigmine 9.5mg, Tamsulosin 0.4mg, Acetylsalicylic acid 100mg | 11 | 10 | parietal bilateral, frontal bilateral, precuneus, anterior cingulum, occ/temp bilateral | 7 | 6 | 47.91 | 48.91 | fatigue (1/sessions) |
| P18 | m | 65 | AD | none | Rivastigmine 9.6mg | 11 | 10 | parietal ext bilateral, frontal bilateral, precuneus, anterior cingulum | 14 | 32 | 60.15 | 70.15 | pressure (1/10 sessions) |
| P19 | f | 68 | AD | condition after right frontal ischemia, condition after hysterectomy, post-ischemic defect in the right parietal white matter | Donepezil 5mg, Levothyroxin 50mg, Acetylsalicylic acid 100mg | 11 | 10 | parietal ext. bilateral, frontal ext. bilateral, precuneus, cing cort, occ/temp bilateral | 14 | 9 | 61.27 | 64.27 | pressure (4/10 sessions) |
| P20 | m | 65 | early-onset AD | none | Escitalopram 5 mg, Rivastigmine 9.5mg | 10 | 10 | parietal bilateral, frontal bilateral, cing cort ext, occ/temp bilateral | 15 | 11 | 65.98 | 69.98 | confusion (1/10 sessions), dizziness (1/10 sessions), pain (1/10 sessions) |
| P21 | m | 52 | early-onset AD | Anxiety Disorder, Depression, insomnia | Levamisole 100mg, Donepezil 5mg | 10 | 10 | parietal bilateral, frontal bilateral, precuneus, occ/temp bilateral | 21 | 12 | 30.35 | 33.35 | none |
| P22 | f | 81 | Senile dementia of AD type | Adjustment disorder | Calcium & Vit.D3 500mg/800 I.E, Donepezil 10mg, Trospium chloride 30mg, Sertraline 100mg, Zofenopril | 12 | 10 | parietal ext bilateral, frontal bilateral, precuneus, anterior cingulum, occ/temp bilateral | 3 | 0 | 33.38 | 35.38 | dizziness (2/10 sessions), gait disturbance (1/10 sessions) |
| P23 | f | 67 | AD | mild hypercholesterolemia | Bisoprolol 5mg, Rivastigmine 9.5mg (then 13.3mg), Simvastatine 40mg | 10 | 10 | parietal bilateral, frontal bilateral, precuneus, anterior cingulum, occ/temp bilateral | 25 | 11 | 26.62 | 31.94 | none |
| P24 | m | 80 | AD | arterial hypertension, benign prostatic hyperplasia, bifascicular block, cAVK I, cerebral atherosclerosis, diabetes mellitus type 2, diabetic polyneuropathy, gastritis, glaucoma, nephrolithiasis, pansinusitis, steatosis hepatis, transverse polyp, vascular leukoencephalopathy | Glimepiride 3mg, Cinnarizine 80mg, Memantine 20mg, Linagliptin & Metformin 2.5/1000mg, Acetylsalicylic acid 100mg | 10 | 10 | parietal bilateral, frontal bilateral, precuneus ext, anterior cingulum, occ/temp bilateral | 4 | 0 | 49.63 | 51.63 | dizziness (1/10 sessions), fatigue (4/10 sessions), pain (1/10 sessions) |
| P25 | m | 75 | mixed dementia | aortic valve insufficiency 1 in aortic aneurysm, arterial hypertension, hypercholesterolemia, path. fasting glucose | Donepezil 10mg, Cinnarizine 80mg | 11 | 10 | parietal bilateral, frontal bilateral, precuneus, anterior cingulum, occ/temp bilateral | 7 | 0 | 58.81 | 54.81 | none |
| P26 | m | 77 | mixed dementia | Aortic valve stenosis, chronic renal insufficiency, condition after thyroidectomy, COPD grade 1-2, diabetes mellitus type 2 | Donepezil 10mg, Atorvastatin 20mg, Levothyroxine 100mcg, Empagliflozin & Metformin 12.5/850 mg, Clopidogrel 100mg, Pentoxifylline | 11 | 10 | parietal bilateral, frontal bilateral, precuneus, anterior cingulum, occ/temp bilateral | 17 | 4 | 84.56 | 84.56 | fatigue (1/10 sessions) |
| P27 | m | 82 | mixed dementia | arterial hypertension, Diabetes mellitus type 2, left ventricular hypertrophy, polyneuropathy, renal insufficiency | Bisoprolol 5mg, Candesartan 32mg, Linagliptin & Metformin 2.5mg, Pioglitazon 15mg, Rivastigmine 9.5mg/24h, Solifenacin 5mg | 12 | 10 | parietal bilateral, frontal bilateral, precuneus, anterior cingulum, occ/temp bilateral | 4 | 4 | 70.56 | 71.56 | dizziness (1/10 sessions) |
| P28 | f | 78 | vascular dementia | Condition after mitral valve reconstruction, condition after total hip replacement, bilateral pneumonia, colitic ulcerosa, monoclonal proteinuria MGUS | Bisoprolol 5mg, Calcium, Vitamin D3**,** Sacubitril 49mg/51 mg, Furosemide 40 mg, Latanoprost 50 mg, Mesalazine 3000mg, Pantoprazole 40mg, Atorvastatin 20mg, Spironolactone, Acetylsalicylic acid 100mg | 12 | 10 | parietal bilateral, frontal ext bilateral, precuneus, anterior cingulum, occ/temp bilateral | 2 | 8 | 58.41 | 54.41 | none |
| P29 | m | 82 | AD | Arterial hypertension, recurrent syncope, bilateral nodular goiter (Hashimoto), iron deficiency anemia, | Cinnarizine 80mg, Enalapril 20mg, Rivastigmine 9.5mg, Atorvastatin 10mg, Acetylsalicylic acid 100mg, Levothyroxine 75mg | 11 | 10 | parietal bilateral, frontal bilateral, precuneus, anterior cingulum, occ/temp bilateral | 4 | 11 | 67.46 | 56.48 | none |
| P30 | m | 56 | atypical AD | Simultanagnosia under investigation | Amlodipine & Atorvastatin 5/10mg, Cinnarizine | 11 | 10 | parietal bilateral, frontal bilateral, precuneus, anterior cingulum, occ/temp bilateral | 14 | 10 | 57.93 | 65.93 | pain (1/10 sessions) |
| P31 | m | 56 | AD | Adjustment disorder, Arterial hypertension, beginning neurodegenerative tauopathy, organic mnestic disorder, Suspected mild cognitive disorder | Candesartan 16mg, Doxycycline 200mg | 12 | 10 | parietal bilateral, frontal bilateral, precuneus, anterior cingulum, occ/temp bilateral | 0 | 0 | 73.44 | 78.44 | pain (1/10 sessions) |
| P32 | m | 74 | AD | Hypertension, condition after ischemic stroke, condition after rec. prostates, condition after PM | Enoxaparin 40mg, Rivaroxaban 20mg (paused) | 12 | 10 | parietal bilateral, frontal bilateral, precuneus, anterior cingulum, occ/temp bilateral | 7 | 6 | 41.93 | 44.93 | pain (1/10 sessions), pressure (1/10 sessions) |
| P33 | m | 57 | MCI with underlying early-onset AD | condition after feverish illness suspected to be caused by African wandering tick | Donepezil 5mg | 11 | 10 | parietal bilateral, frontal bilateral, precuneus, anterior cingulum | 15 | 4 | 74.56 | 68.56 | none |
| P34 | f | 79 | Senile dementia of AD type | Anxious agitated mood, Cerebral deterioration, Gait disturbance | Donepezil 10mg, Mirtazapine 10mg, Pramipexole 0.35mg | 11 | 10 | parietal bilateral, frontal bilateral, precuneus, anterior cingulum, occ/temp bilateral | 27 | 29 | 70.63 | 69.63 | none |
| P35 | m | 70 | AD | hypercholesterolemia, latent diabetes mellitus, mild extracranial arteriosclerosis | dietery supplements only | 11 | 10 | parietal bilateral, frontal bilateral, precuneus, anterior cingulum, occ/temp bilateral | 51 | 11 | 57.08 | 49.08 | dizziness (1/10 sessions), fatigue (3/10 sessions), pain (2/10 sessions) |
| P36 | m | 65 | Senile dementia of AD type, mixed | condition after ACMS insult, hypertension | Memantine 20mg, Sertraline 100mg, Acetylsalicylic acid 100mg | 11 | 10 | centroparietal bilateral, frontal bilateral, precuneus ext, anterior cingulum | 3 | 3 | 16.74 | 19.74 | none |
| P37 | f | 72 | AD | fract. subcapit hum dex, osteoporosis, posttraumatic omarthrosis right | Ginkgo Biloba, Phosphatidylserine, Vitamin B, E | 11 | 10 | parietal bilateral, frontal bilateral, precuneus, anterior cingulum, occ/temp bilateral | 11 | 7 | 46.46 | 46.46 | fatigue (1/10 sessions), pain (2/10 sessions), pressure (2/10 sessions) |
| P38 | m | 61 | AD | Autoimmune thyroid disease of the Hashimoto type, Depressive disorder, Post-polytrauma condition | Atorvastatin 40mg, Donepezil 5mg, Duloxetine 60mg, Levothyroxine 150mg, Memantine 5mg, Selenium 200 NE, Cholecalciferol | 11 | 10 | parietal bilateral, frontal bilateral, precuneus, anterior cingulum, occ/temp bilateral | 3 | 0 | 64.86 | 44.86 | none |
| P39 | f | 81 | Senile dementia of AD type | removal of the appendix, ovaries and abdominal omentum (tumor), squamous epithelium about 10 years ago, stroke 23 years ago | Actavis, Cadesartan, Donepezil | 10 | 10 | parietal bilateral, frontal bilateral, precuneus, anterior cingulum, occ bilateral | 4 | 0 | 63.79 | 61.79 | pain (1/10 sessions) |
| P40 | f | 74 | AD | condition after spondylolisthesis operation, hypertension, dyslipidemia, gastroesophagal reflux, hiatal hernia, hypothyriodism | Atorvastatin 40mg, Calcium Carbonate 1000mg, Donepezil 5-10 mg, Mebeverine, Levothyroxine 125, Enalapril 10mg, Esomeprazole 15mg | 10 | 10 | parietal bilateral, frontal bilateral, precuneus, anterior cingulum, occ/temp bilateral | 1 | 0 | 57.21 | 66.21 | fatigue (3/10 sessions) |
| P41 | m | 82 | MCI | condition after anaphylaxis to insect bite, condition after traumatic brain injury 2019, macular degeneration on both sides, multisegmental neuroforamen stenosis C3-C7, Prostate hypertrophy following TURP | Finasteride, Cinnarizine, Omega-3 fatty acid supplement 1400, Lutein, Zeaxanthin, Rivastigmine, Acetylsalicylic acid | 11 | 10 | parietal bilateral, frontal bilateral, precuneus, anterior cingulum, occ/temp bilateral | 3 | 0 | 69.97 | 77.97 | confusion (1/10 sessions), pain (1/10 sessions), pressure (5/10 sessions) |
| P42 | m | 80 | Incipient Dementia | Depression, Onychomycosis/ Dr Postl, hypertension, mild CNI (Crea 1.2-1.4), right omarthrosis & supraspinatus tendon rupture without surgery. Stp TU right OS, KTEP II, HTEP right | Amlodipin/Valsartan 80/5, Atorvastatin 20mg plus, Cinnarizine 80mg, Donepezil 10mg, Amlodipine & Valsartan & Hydrochlorothiazide 160/5/12.5, Ezetimib, Cholecalciferol, Dabigatran 150, Sertralin 50mg | 11 | 10 | parietal bilateral, frontal bilateral, precuneus, anterior cingulum, occ/temp bilateral | 3 | 0 | 63.43 | 62.43 | pressure (1/10 sessions) |
| P43 | f | 57 | atypical AD | Condition after thyroidectomy, condition after closure of a Patent foramen ovale | Levothyroxine 75mg + 100mg, Memantine 10mg | 11 | 10 | parietal bilateral, frontal bilateral, precuneus, anterior cingulum, occ/temp bilateral | 41 | 17 | 37.70 | 30.70 | none |
| P44 | f | 78 | mixed dementia of AD type | DP C3/4 and C4/5 without root contact, extension malposition of the cervical spine, vascular leukoencephalopathy and leukoaraiosis, selective IgM deficiency | Ezetimibe/Simvastatin 10/20mg, Domperidone 10mg, Pantoprazole 40mg, Rivastigmine 1.5mg, Piracetam 40mg, Acetylsalicylic acid 100mg | 11 | 10 | parietal bilateral, frontal bilateral, precuneus, anterior cingulum, occ/temp bilateral | 4 | 0 | 45.51 | 49.51 | none |
| P45 | f | 81 | amnestic MCI | Arterial hypertension, ChE, Diabetes mellitus type 2, Fatty liver of unclear etiology, Generalized anxiety disorder, persistent anxious depression, TE | Candesartan 8-12mg, Bisoprolol 2.5mg, Metformin 500mg, Donepezil 5mg, Lansoprazole 40mg, Acetylsalicylic acid 100mg, Ursodeoxycholic acid 500mg | 11 | 10 | parietal bilateral, frontal bilateral, precuneus, anterior cingulum | 4 | 0 | 55.48 | 66.48 | dizziness (1/10 sessions), fatigue (1/10 sessions), pain (1/10 sessions) |
| P46 | f | 78 | mixed dementia | Depression, Diabetes mellitus type 2, Hypertension | Berberine, Policosanol, Empagliflozin /Linagliptin 25/5mg, Mangiferin, Bisoprolol 5mg, Cholecalciferol, Spermedine memory, Ginkgo Biloba 240mg | 11 | 10 | parietal bilateral, frontal bilateral, precuneus, anterior cingulum | 0 | 0 | 48.92 | 52.92 | nausea (1/10 sessions) |
| P47 | f | 71 | AD | vertigo, no other diagnoses reported | Clopidogrel 75mg, Donepezil 5mg, Rosuvastatin 5mg, Vitamin D3, Vitamin E | 12 | 10 | parietal bilateral, frontal bilateral, precuneus, anterior cingulum, occ/temp bilateral | 1 | 0 | 32.26 | 29.26 | confusion (1/10 sessions), fatigue (1/10 sessions) |
| P48 | m | 75 | MCI in context of AD | essential hypertension, Hypothyroidism, malignant neoplasm of the prostate, mild depressive episode | Cilostazol 0.4mg, Levothyroxine 75 mikrog, Rivsatigmine 13.3mg/h, Bupropion 150mg | 19 | 10 | parietal bilateral, frontal bilateral, precuneus, anterior cingulum, occ/temp bilateral | 10 | 6 | 74.60 | 75.60 | confusion (2/10 sessions), fatigue (3/10 sessions) |
| P49 | m | 82 | mixed dementia | none | Memantine 10mg, Vitamin D3 | 11 | 10 | parietal bilateral, frontal bilateral, precuneus, anterior cingulum, occ/temp bilateral | 4 | 0 | 64.38 | 67.38 | pain (1/10 sessions) |
| P50 | f | 75 | vascular dementia | essential hypertension, depressive episode, disorder of purine and pyrmidine metabolism, glaucoma, heart failure, hyperlipidemia, monoclonal gammopathy of undetermined significance, ulcerative colitis | Bisoprolol 5mg, Calcium, Vitamin D3, Sacubitril /Valsatran 49mg/51mg, Furosemide 40mg, Latanoprost 50mg, Omega-3 fatty acids 3000mg, Sertralin 50mg, Atorvastatin 20mg, Spironolactone, Acetylsalicylic acid 100mg | 11 | 10 | parietal bilateral, frontal bilateral, precuneus, anterior cingulum, temporal bilateral | 4 | 0 | 54.23 | 47.23 | dizziness (2/10 sessions), fatigue (4/10 sessions), pressure (1/10 sessions) |
| P51 | f | 54 | early-onset AD | depressive episode, exhaustion syndrome (in 2021) | Rivastigmine 3mg | 14 | 10 | parietal bilateral, frontal bilateral, precuneus, anterior cingulum | 9 | 92 | 35.84 | 43.84 | fatigue (2/10 sessions) |
| P52 | f | 73 | MCI | arterial hypertension, migraine without aura | Amlodipin 5mg, Cinnarizine 80mg, Escitalopram 10mg, Eplerenon 25mg, Guanfacine 2mg | 11 | 10 | parietal bilateral, frontal bilateral, precuneus, anterior cingulum | 6 | 0 | 92.58 | 100.58 | dizziness (1/10 sessions), pain (2/10 sessions) |
| P53 | f | 67 | Senile dementia of AD type | none | Donepezil 5mg | 10 | 10 | parietal bilateral, DLPFC bilateral, precuneus, para/hippo/temp/occ bilateral | 26 | 26 | 68.84 | 65.84 | fatigue (1/10 sessions), pain (2/10 sessions), pressure (2/10 sessions) |
| P54 | f | 66 | AD | none | Donepezil (discontinued during therapy pause), Memantine 29mg | 32 (hospitalized in-between) | 12 | parietal bilateral, frontal bilateral, precuneus, anterior cingulum, occ/temp bilateral | 3 | 0 | 56.62 | 60.62 | fatigue (1/12 sessions) |
| P55 | f | 78 | Senile dementia of AD type | art. hypertension, finger polyarthrosis, condition after HE, chronic dorsolymbalgia, condition after CHE, condition after hemithyroidectomy, severe torsional scoliosis lumbar, spondylarthrosis + NFS lumbar spine, disc protrusions lumbar multiseg., recess stenosis L5/S1, solid cyst. kidney TU left, atherosclerosis, monoclonal gammopathy, leukariasis paraprotenemia IgG Lamba | Acetylcysteine 600mg, Calcium + Vitamin D3 500mg/800ie, Donepezil 10mg, Fentanyl 25mcg/h, Latanoprost 50mcg/ml, Vitamin B, Ramipril 2.5mg, Ferrous sulfate 80mg, Acetylsalicylic acid 100mg, Betahistine 50mg | 11 | 10 | parietal bilateral, frontal bilateral, precuneus, anterior cingulum | 3 | 6 | 62.10 | 70.10 | fatigue (3/10 sessions), pain (2/10 sessions) |
| P56 | m | 74 | vascular dementia, Multi-infarct syndrome | Arterial hypertension, Mixed hyperlipidemia, mild hemiparesis on the right, organic brain syndrome, Suspected cerebral amyloid angiopathy | Amlodipin 5mg, Candesartan Hydrochlorothiazide 16mg, Rosuvastatin 40mg, Dalteparin 5000 units, Itraconazole 1mg, Pantoprazole 40mg, Acetylsalicylic acid 100mg | 14 | 10 | centroparietal bilateral, DLPFC bilateral, precuneus ext., anterior cingulum ext. | 0 | 0 | 47.07 | 58.07 | dizziness (1/10 sessions), pain (1/10 sessions) |
| P57 | m | 81 | mixed dementia | after years of extensive C2 consumption DD Korsakow Dementia, brain atrophy, hydrocephalus e vacuo, hypoacusis | Rivastigmine 9.5 mg | 11 | 8 | parietal bilateral, frontal/DLPFC bilateral, precuneus, anterior cingulum, occ/temp bilateral | 21 | 17 | 66.24 | 58.56 | pain (1/8 sessions) |
| P58 | m | 78 | mixed dementia | Hypercholesterolemia, Mild arteriosclerosis of the carotid arteries, non-stenosing coronary sclerosis, Ocular myasthenia gravis, Sick sinus syndrome of the bradycardia type, Vit D3 deficiency | Ginkgo biloba80mg,  Clopidogrel 75mg, Donepezil 5mg, Midodrine 5mg, Azathioprine, Pyridostigmine 60mg, Cholecalciferol, Quetiapine 25mg, Rosuvastatin/ Amlodipine 5/10mg | 16 | 10 | parietal bilateral, frontal bilateral, precuneus, anterior cingulum, occ/temp bilateral | 0 | 0 | 41.26 | 48.26 | none |

*Note.* The mentioned adverse events include those reported over the course of the whole treatment with their total frequencies counted over all sessions.
